# Systemic lupus erythematosus activity and beta two microglobulin levels

**DOI:** 10.1590/1516-3180.2014.1324703

**Published:** 2014-05-28

**Authors:** Thelma Larocca Skare, Kellen Ferri, Marcela Aimone Santos

**Affiliations:** I MD, PhD. Associate Professor, Discipline of Rheumatology, Faculdade Evangélica do Paraná, and Head of Rheumatology Unit, Hospital Universitário Evangélico de Curitiba, Curitiba, Paraná, Brazil; II F Medical Student. Faculdade Evangélica do Paraná, Curitiba, Paraná, Brazil

**Keywords:** Lupus erythematosus, systemic, beta 2-microglobulin, Inflammation, Blood sedimentation, Acute-phase reaction, Lúpus eritematoso sistêmico, Microglobulina-2 beta, Inflamação, Sedimentação sanguínea, Reação de fase aguda

## Abstract

**CONTEXT AND OBJECTIVE:**

Systemic lupus erythematosus (SLE) is an autoimmune disease with a cyclical clinical course. Evaluation of the clinical activity of this disease is important for choosing the correct treatment. The objective of this study was to analyze the value of beta-2 microglobulin (β2M) serum levels in determining SLE clinical activity.

**DESIGN AND SETTING:**

Cross-sectional analytical study conducted at the rheumatology outpatient clinic of a private university hospital.

**METHODS:**

129 SLE patients were studied regarding disease activity using SLEDAI (SLE Disease Activity Index) and cumulative damage using SLICC ACR (SLE International Collaborating Clinics/American College of Rheumatology Damage Index for SLE). At the same time, the β2M serum level, ESR (erythrocyte sedimentation rate), anti-dsDNA (anti-double-stranded DNA) and C3 and C4 complement fractions were determined.

**RESULTS:**

β2M levels correlated positively with SLEDAI (P = 0.02) and ESR (P = 0.0009) and negatively with C3 (P = 0.007). Patients who were positive for anti-dsDNA had higher β2M serum levels (P = 0.009).

**CONCLUSION:**

β2M levels are elevated in SLE patients with active disease.

## Abstract (pt)

**CONTEXTO E OBJETIVO:**

O lúpus eritematoso sistêmico (LES) é uma doença autoimune com curso clínico flutuante. Determinar a atividade clínica da doença é importante na escolha do tratamento correto. O objetivo deste estudo foi o de analisar se os níveis séricos de beta-2 microglobulina (β2M) têm valor na determinação da atividade clínica do LES.

**TIPO DE ESTUDO E LOCAL:**

Estudo transversal, analítico, realizado no ambulatório de reumatologia de um hospital universitário particular.

**MÉTODOS:**

129 pacientes com LES foram estudados para atividade da doença pelo SLEDAI (SLE Disease Activity Index) e para dano cumulativo pelo SLICC (SLE International Collaborating Clinics/American College of Rheumatology Damage Index for SLE). Simultaneamente foram determinados os valores séricos de β2M, das frações C3 e C4 do complemento, VHS (velocidade de hemossedimentação) e anti dsDNA (DNA de dupla fita).

**RESULTADO:**

Encontrou-se correlação positiva entre níveis de β2M com o SLEDAI (P = 0,02), com VHS (P = 0,0009) e correlação negativa com níveis de C3 (P = 0,007). Pacientes com presença de anti dsDNA tinham níveis mais altos de β2M (P = 0,009).

**CONCLUSÃO:**

Níveis de β2M estão elevados em pacientes de LES com doença ativa.

## INTRODUCTION

Systemic lupus erythematosus (SLE) is an autoimmune disease with periods of flares and remissions.^1^ The treatment for SLE accompanies the degree of disease activity and, thus, determining this activity level is very important, even though difficult. Commonly-used inflammatory markers such as sedimentation rate (ESR) and C-reactive protein are nonspecific and unreliable. 1 On the other hand, composite measurements such as the SLE Disease Activity Index (SLEDAI) and the British Isles Lupus Assessment Group index (BILAG),^2^ which combine laboratory and clinical findings, are time-consuming and not easily applicable in daily practice.

Beta-2 microglobulin (β2M) is a light-chain subunit of class I human leukocyte antigen (HLA) that exists on the cell membrane of all nucleated cells.^3^ It sheds from the membrane and is measurable in the bloodstream, where its levels are elevated in diseases with high lymphoproliferative activity.^3^ Some authors have noted increased plasma and/or urine β2M levels in Sjögren's syndrome, rheumatoid arthritis and SLE,^4^ and have proposed that measuring its levels might be useful as an activity marker. 

## OBJECTIVE

The present analysis was undertaken to further examine the relationship between β2M and lupus activity, along with the cumulative damage in a sample of Brazilian patients.

## METHODS

This was a cross-sectional analytical study that was approved by the local Research Ethics Committee. All subjects signed an informed consent statement.

We included 129 patients from a single tertiary center, who were all over 18 years of age and met at least four of the classification criteria of the American College of Rheumatology for SLE.^5^ Patients with overlapping features, creatinine above 1.1 mg/dl, previous history of lymphoproliferative disorders, pregnancy or associated infections were excluded. Demographic and clinical data were obtained through chart review. 

All individuals underwent measurement of SLEDAI^2^ and SLICC/ACR^6^ (Systemic Lupus Erythematosus International Collaborating Clinics/American College of Rheumatology Damage Index for SLE) along with measurements of β2M, C3, C4, ESR (erythrocyte sedimentation rate), CRP (C-reactive protein), hemoglobin (Hb) levels and anti-dsDNA (anti-double-stranded DNA). 

β2M measurements were made in serum by means of chemiluminescence (Immulite 2000, Diagnostic Products Corporation, USA*)*, with normal values ranging from 604 to 2786 ng/ml; ESR was measured using the Westergreen method (normal value < 8 mm); CRP was measured by means of immunoturbidimetry (normal values < 0.50 mg/dl); and Hb was measured using an automated method (normal values of 12.2-18.1 g/dl). C3 and C4 were evaluated in fasting serum by means of immunoturbidimetry (normal values for C3 = 82-170 mg/dl; and for C4 = 12-36 mg/dl). Anti-dsDNA was analyzed by means of indirect immunofluorescence using *Crithidia lucilae* as the substrate (Immunoconcept, Alka, São Paulo, Brazil).

The data were compiled in frequency tables. The central trend was expressed as the mean and standard deviation for parametric data and as the median and interquartile range (IQR) for nonparametric data. Correlation analyses were conducted using the Spearman and Pearson tests, according to sample distribution. An association analysis on β2M levels in the presence of anti-dsDNA was done using the Mann-Whitney test. The GraphPad Prism software, version 4.0, was used for calculations. The significance level was taken to be 5%.

## RESULTS

In this sample, 3.1% were male and 96.9% were female, with a mean age of 40.1 ± 11.3 years and median disease duration of 8.0 years. Regarding the cumulative clinical profile: 67.1% patients had photosensitivity; 60.1% arthritis; 43.3% oral ulcers; 39.8% butterfly rash; 36.3% glomerulonephritis; 32.1% leucopenia; 9.0% pericarditis; 8.3% hemolytic anemia; 8.3% seizures; 6.9% discoid lesions; 5.5% pleuritis; 6.2% thrombocytopenia; and 3.4% psychosis.

In this population, 54.4% were using glucocorticoids; 71.2% antimalarials; 17.8% azathioprine; 15.8% methotrexate; 5.9% mycophenolate mofetil; 1.9% cyclophosphamide; 0.9% cyclosporine and 0.9% thalidomide. 

SLEDAI ranged from 0 to 14 (median 0.0; IQR = 0.0-2.0); and SLICC ranged from 0 to 9 (median = 1.0; IQR = 0.0-2.0). The median ESR was 25.0 mm (IQR = 9.0-44.5 mm); the median C3 level was 111.0 mg/dl (IQR = 92.8-131.1 mg/dl) and median C4, 19.0 mg/dl (IQR = 13.8-23.7 mg/dl). The median Hb level was 13.0 g/dl (IQR = 12.3-14.0) and median CRP was 2.6 mg/dl.

In 28.6% of the patients, anti-dsDNA was positive; β2M ranged from 1120 to 4943 ng/ml (median of 2045 ng/ml; IQR from 1679-2591 ng/ml). The correlations of β2M with SLEDAI, SLICC, complement, hemoglobin, C-reactive protein and ESR can be seen in Table 1. The median value for β2M in patients with positive anti-dsDNA was 2167 ng/ml (1829-3003 ng/ml), while in those without anti-dsDNA the median was 1950 ng/ml (1600-2307 ng/ml; P = 0.009).

**Table 1:** Result from correlating β2 microglobulin levels with the Systemic Lupus International Collaborating Clinics/American College of Rheumatology (SLICC/ACR) index, systemic lupus erythematosus disease activity index (SLEDAI), complement, hemoglobin, C-reactive protein and sedimentation rate

	R	95% confidence interval	P
SLICC/ACR	-0.05	-0.23 to 0.12	0.50
SLEDAI	0.20	0.02 to 0.36	0.02
C3 (mg/dl)	-0.23	-0.39 to -0.05	0.007
C4 (mg/dl)	-0.12	-0.29 to 0.05	0.17
Sedimentation rate (mm)	0.28	0.11 to 0.44	0.0009
Hemoglobin (g/dl)	-0.26	-0.42 to -0.09	0.002
C-reactive protein	0.21	0.03 to 0.37	0.014

The correlation of SLEDAI with C3 showed P = 0.01 (R = -0.21; 95% confidence interval, CI = -0.37 to -0.03); with ESR, P = 0.04 (R = 0.17; 95% CI = 0.001 to 0.34); and with CRP, P = 0.28 (R = 0.09; 95% CI = -0.08 to 0.26). Patients with anti-dsDNA had a median SLEDAI of 4.0, while those without it had a median value of 1.0 (P = 0.01).

## DISCUSSION

β2M is a small protein that has an amino acid sequence related to the constant parts of heavy and light chains of immunoglobulins. 7 It locates on the surface of all nucleated cells and its bestcharacterized function is to interact with and stabilize the tertiary structure of the MHC class 1 chain.7 β2M is normally found in serum, urine and other body fluids and is almost exclusively catabolized within the kidney; 95% to 100% of circulating β2M is eliminated through glomerular filtration.7

The reason why it has been found to be elevated in SLE patients is unknown. Some authors^4^ believed that this might result from the increased turnover of lymphocytes seen in this disease. Autoantibodies directed against β2M that present lymphocytotoxic activity have also been reported in SLE.^8^ Since immune complexes formed by β2M and anti-β2M have a larger size, they cannot be filtered by the kidney, thus raising the serum levels of β2M and giving another explanation for this elevation.

Walters et al.^9^ detected higher levels of serum β2M in rheumatoid arthritis patients and an even higher concentration in synovial fluids, which suggested that there is intra-articular production of this protein in this disease. Interestingly, in studying the possible associations between β2M and specific lupus manifestations, Kim et al.^4^ found that the serum levels were higher in patients with serositis, oral ulcers and glomerulonephritis but not in those with arthritis. Experimental studies^10^ on β2M-deficient lupus mice showed that a lack of β2M caused dissociation in the clinical expression of the disease, with aggravation of skin disease and amelioration of nephritis. This divergence of disease in the skin and kidneys of β2M-deficient mice suggests that target organs may respond in different ways to the autoimmune process in this context.

Another study on 26 lupus patients, by Hermansen et al.,^11^ found that serum β2M showed correlations with cytokines such as interleukin (IL)-6, IL-8, IL-10 and IL-18, and with serum interferon-Î±. These authors believed that the increased β2M levels in active SLE reflected the overall immunological activity.

In the present study, we found that ESR, CRP, C3 and SLEDAI were associated with β2M, thus showing that its level was elevated in cases of active disease. This result is similar to the findings of Evrin and Ström^8^ in 23 Swedish lupus patients, in whom they found a positive correlation with ESR and a negative association with hemoglobin levels. Moreover, Kim et al.^4^ studying Korean lupus population found a negative correlation between β2M and C3 and a positive correlation between β2M and SLEDAI, as we did. In a disease such as SLE, with great influence of genetic background, our results showed that the levels of β2M in lupus activity in a sample of Brazilian population follow the same pattern as others. Furthermore, we did not find any association of β2M with SLE cumulative damage measured by SLICC/ACR, which had not been studied previously.

We also showed that, in this sample, SLEDAI correlated with ESR, C3 and anti-dsDNA, which raises the question of the additional value in measuring β2M. It needs to be noted that in this study we excluded patients with associated infections, which are a cause of great confusion in ESR interpretation. Also, C3 consumption and presence of anti-dsDNA are associated with specific lupus manifestations. Membranous glomerulonephritis (or class 5 glomerulonephritis) is a typical example in which the lupus manifestation may appear with a normal complement and without anti-dsDNA.^12^ ^,^ ^13^ In these situations, assaying for β2M may help to determine disease activity.

## CONCLUSIONS

Our findings provide confirmation that β2M levels are elevated in cases of active lupus, which thus may help in determining the degree of disease activity. Further studies are needed in order to understand the role of this protein in the pathophysiological process of lupus.
